# Measuring women’s experiences of maternity care: protocol for a systematic review of self-report survey instruments

**DOI:** 10.1186/s13643-019-1261-8

**Published:** 2020-01-06

**Authors:** Claire Beecher, Richard Greene, Laura O’Dwyer, Ethel Ryan, Mark White, Michelle Beattie, Declan Devane

**Affiliations:** 1 School of Nursing and Midwifery, National University of Ireland, Galway, Ireland; 2 National Perinatal Epidemiology Centre, Department of Obstetrics and Gynaecology, Cork University Maternity Hospital, Cork, Ireland; 3 Department of Measurement, Evaluation, Statistics & Assessment, Boston College, Chestnut Hill, Massachusetts USA; 4 Department of Paediatrics, University Hospital Galway, Galway, Ireland; 5 Programme for Health Service Improvement, Health Service Executive, Dublin, Ireland; 6 Department of Nursing, University of the Highlands and Islands, Inverness, Scotland; 7 Health Research Board - Trials Methodology Research Network (HRB-TMRN), Galway, Ireland; 8 Evidence Synthesis Ireland, Galway, Ireland

**Keywords:** Midwifery, Maternity, Measurement, Experiences of care, Quality care, Systematic review protocol, Instruments, Questionnaires, Surveys

## Abstract

**Background:**

The use of survey instruments to measure women’s experiences of their maternity care is regarded internationally as an indicator of the quality of care received. To ensure the credibility of the data arising from these instruments, the methodological quality of development must be high. This paper reports the protocol for a systematic review of self-report instruments used to measure women’s experiences of their maternity care.

**Methods:**

Citation databases CINAHL, Ovid MEDLINE and EMBASE will be searched from 2002 to 2018 using keywords including women, experience, maternity care, questionnaires, surveys, and self-report. Citations will be screened by two reviewers, in two rounds, for inclusion as per predetermined inclusion and exclusion criteria. Data extraction forms will be populated with data, extracted from each study, to evaluate the methodological quality of each survey instrument and the criteria for good measurement properties using quality criteria. Data will also be extracted to categorise the items included in each survey instrument. A combination of a structured narrative synthesis and quantitate summaries in tabular format will allow for recommendations to be made on the use, adaptation and development of future survey instruments.

**Discussion:**

The value of survey instruments that evaluate women’s experiences of their maternity care, as a marker of quality care, has been recognised internationally with many countries employing the use of such instruments to inform policy and practice. The development of these instruments must be methodologically sound and the instrument itself fit for the purpose and context in which it is used. This protocol describes the methods that will be used to complete a systematic review that will serve as a guide for choosing the most appropriate existing instruments to use or adapt so that they are fit for purpose, in addition to informing the development of new instruments.

**Systematic review registration:**

PROSPERO CRD42018105325

## Background

The measurement of quality healthcare from the service users’ perspective is a crucial element in the application of quality assurance and improvement processes [1, 2]. Much of the quantitative measurement of the quality of care from the service user’s perspective has focused on two aspects, i.e. satisfaction and experiences of care. The use of survey instruments to measure satisfaction with healthcare dates back to the 1950s [3]. However, the adequacy of satisfaction as a measure of quality has been questioned because it is indirectly related to the quality of care received [4, 5]. Satisfaction is also, generally, rated high by service users regardless of the quality of care that has been received, and has been attributed to a reluctance to criticise caregivers, as well as service users valuing what is known, or available, to them [6, 7]. Given these limitations, the use of survey instruments that focus on the measurement of experiences of care, as an indicator of quality, has become more prominent. In contrast to satisfaction, experiences of care focus on accounts of the care received [8, 9]. Survey instruments that measure experiences of care minimise the need for respondents to make evaluations on their care and focus on the reporting of what did or did not happen [9].

A reliance on the measurement of experiences of care to inform policy and practice is evident within maternity services [10–12]. There are, however, many challenges to measuring women’s experiences of their maternity care. Maternity care is complex, encompassing numerous services at various time points, with a wide variety of professions and professionals along a temporal care continuum [13]. Furthermore, models of maternity care vary significantly between jurisdictions. The complex nature of maternity care coupled with the variances in services internationally has led to a proliferation of instruments that each seek to gather data on the quality of various aspects of these services from the perspective of women as service users [4].

Measurement of women’s experience of maternity care requires robust instruments. Clinicians, managers, policymakers, and researchers must have access to the processes of development and measurement properties of these instruments when using or adapting existing instruments or developing new instruments to ensure that the resulting data, often used to direct policy and practice, is credible [14, 15]. The aim of this review is to systematically review and critically appraise self-report survey instruments measuring women’s experiences of their maternity care. The results of this review will serve as the basis of future research on the use, adaptation and development of self-report survey instruments internationally to measure women’s experiences of their maternity care. For example, within the republic of Ireland specifically, these results will inform the development of the National Maternity Experience Survey (see https://yourexperience.ie/maternity/about-the-survey/).

The objectives of the review are to

1. Identify self-report survey instruments that are available internationally to measure women’s experiences of their maternity care;
2. Categorise items included within each survey instrument;
3. Evaluate the methodological quality of each survey instrument; and
4. Evaluate the criteria for good measurement properties using quality criteria.

## Methods and design

### Design and registration

The review protocol was submitted to the International Prospective Register of Systematic Reviews (PROSPERO) on 14 August 2018 (No. CRD42018105325). The protocol has been developed in accordance with the Preferred Reporting Items for Systematic Reviews and Meta-Analysis Protocols (PRISMA-P) statement [16] and the completed review will be reported in accordance with the PRISMA guidelines [17].

### Search strategy

An extensive literature review will be performed and refined using the following citation databases: CINAHL, OVID Medline and Embase. Searching will be limited to literature published from 2002 onwards. This is based on a literature search by Messent [18] who found that up until this point, no published maternity survey instruments had been validated.

An example of a complete search to be carried out is included in Table 1. The search strategy was developed iteratively based on the authors experience (including experienced clinicians and systematic reviewers) and a review of strategies in related reviews. The strategy was then tested in three databases and revised to achieve the best balance between sensitivity and specificity of retrieved citations. The search strategy included in Table 1 has been formulated for use in the CINAHL database and will be adapted to respective databases.

**Table 1:** Search strategy for CINAHL database

Search #	Search string
1	(women*) N5 (experience* OR perspective* OR perception* OR perceiv* OR view*)
2	(patient*) N5 (experience* OR perspective* OR perception* OR perceiv* OR view*)
3	#1 OR #2
4	(MH “Prepregnancy Care”) OR (MH “Prenatal Care”) OR (MH “Postnatal Care”) OR (MH “Obstetric Care”) OR (MH “Perinatal Care”) OR (MH “Patient Care”) OR (MH “Intrapartum Care”
5	(MH “Childbirth”)
6	(MH “Maternal Health Services”) OR (MH “Obstetric Service”) OR (MH “Maternal-Child Care”) OR (MH “Nurse-Midwifery Service”) OR (MH “Midwifery Service”)
7	“maternity care” OR childbirth OR “maternity services”
8	“antenatal care” OR “prenatal care” OR “intrapartum care” OR “postnatal care” OR “obstetric care”
9	“antenatal services” OR “prenatal services” OR “intrapartum services” OR “postnatal services” OR “obstetric services”
10	#4 OR #5 OR #6 OR #7 OR #8 OR #9
11	(MH “Process Assessment (Health Care)”) OR (MH “Health Care Delivery”)
12	“health care measurement” OR “maternity care measurement”
13	(MH “Structured Questionnaires”|) OR (MH “Open-Ended Questionnaires”) OR (MH “Questionnaires”)
14	(MH “Surveys”)
15	(MH “Patient-Reported Outcomes”)
16	questionnaire* OR survey* OR measure* OR tool* OR assessment OR validation OR “patient reported” OR evaluat* OR “self-report”
17	#11 OR #12 OR #13 OR #14 OR #15 OR #16
18	#3 AND #10 AND #17

MH represents CINAHL headings. N5 represents the number of words that could appear between keywords/phrase

### Data screening

Once all database searches have been completed, citations will be exported to the reference manager software Endnote X7 and duplicates removed. Remaining citations will then be exported to Covidence [19] and screened by two reviewers for inclusion using predetermined inclusion and exclusion criteria, set out in the following section. The first round of screening will assess eligibility based on title and abstract. Round two of screening will assess the eligibility of all remining studies based on a full-text review. All abstracts and full texts will be sourced as necessary. Disagreements will be resolved through consensus with a third reviewer. Reasons for exclusion decisions at full-text (round 2) will be documented. References of included papers will be analysed for additional literature on the theoretical, empirical and psychometric development of instruments not identified in the original searches.

### Inclusion and exclusion criteria

#### Inclusion criteria

1. Literature that describes the theoretical or empirical development, or tests the psychometrics, of self-report instruments that measure women’s experiences of their maternity care;
2. Literature that focuses on self-report instruments that measure women’s experiences of their maternity care from the perspective of women, rather than staff, families or others;
3. English language;
4. Primary research;
5. Literature that focuses on women’s perceptions or views on their care are to be included as often these terms are used interchangeably with ‘experiences’;
6. Literature that focuses on the measurement of women’s experiences of their entire maternity care process (from conception up to ten days postpartum), rather than one temporal aspect of care specifically, e.g. antenatal care; and
7. Literature that focuses on the measurement of experiences of maternity care as received by women in general, rather than a focus on participants by specific demographics, e.g. teenage pregnancy.

#### Exclusion criteria

1. Case reports and series, systematic reviews or meta-analysis;
2. Literature that focuses on indirect evidence of measurement properties of an instrument; for example, if an instrument is being used within a randomised controlled trial or alternative study, or if the instrument is being used as part of the validation process of an alternative instrument. This exclusion criterion is based on the recommendation of Terwee et al. [20] who suggest that not only is the process of sourcing this literature difficult but it is also a challenge to interpret the evidence provided in terms of validity and responsiveness as no hypothesis about these aspects of the instruments are formulated and tested in such studies;
3. Literature that focuses on instruments that measure women’s level of satisfaction with their care, rather than their actual experience of that care, as per the methodological limitations with satisfaction as discussed earlier in this paper;
4. Literature that focuses on care received from a specific profession, e.g. midwives, as opposed to a measurement of women’s experiences of their maternity care in general;
5. Literature that focuses on brief versions of full instruments that have been reported elsewhere; and
6. Childbirth experiences that merit specific consideration, for example stillbirths. These experiences require more specific approaches based on the needs and experiences of women in these groups.

### Data extraction process

A prespecified data extraction form will be designed and populated with data extracted from each study. This form will be piloted on three studies independently by two reviewers and compared to ensure consistency in the interpretation of the data being extracted and modified as required. Based on the expectation that some instruments will be the focus of multiple studies, these studies will be grouped together to reduce data extraction duplication [21]. Where the same data has been reported in more than one place, the more comprehensive version will be included.

A stepped approach will be employed to facilitate the extraction of data. Double data extraction will commence with both reviewers independently analysing 10% of the overall number of studies for inclusion in the review. The results of this initial analysis will be compared, and errors resolved through discussion. If significant discrepancies (> 10% of all data items extracted per study) are apparent, then double data extraction will continue for a further 10% of studies. The results will again be compared, and errors discussed. If there are still significant discrepancies at this stage of the analysis, double data extraction will continue for all studies for inclusion in the review. If significant discrepancies are not apparent at this stage, each reviewer will take 50% of the remaining studies and extract data independently. Disagreements will be resolved through consensus with a third reviewer.

### Evaluation of methodological quality and quality of results

The COnsensus-based Standards for the selection of health Measurement Instruments (COSMIN) steering committee have developed a guideline for systematic reviews of patient-reported outcome measures (PROM) [22]. This guideline, adapted as necessary to suit this review, will be used to guide the evaluation of the measurement properties of each included study.

Each measurement property will be evaluated via a three-step process: (1) evaluate the methodological quality of each included study, (2) evaluate the criteria for good measurement properties using quality criteria and (3) summarise the evidence.

1. *Evaluate the methodological quality of each included study*: This COSMIN Risk of Bias checklist [23, 24] is an adaptation of the original COSMIN checklist [25]. The Risk of Bias checklist has been developed specifically for use in systematic reviews of PROMs to assess the risk of bias of studies on measurement properties.
  The checklist contains one box on development and nine boxes for measurement properties (content validity, structural validity, internal consistency, cross-cultural validity/measurement invariance, reliability, measurement error, criterion validity, hypothesis testing for construct validity and responsiveness) [23]. The COSMIN expert group reached international consensus on the taxonomy, terminology and definitions of these measurement properties; full explanations are available from Mokkink et al. [26].
  Each box contains between 3 and 35 items and applicable items will be scored for each included study based on a four-point rating scale (i.e. ‘inadequate’, ‘doubtful’, ‘adequate’ or ‘very good’). An overall score for the methodological quality of a given measurement property is determined by the lowest rating that is assigned to the items within a given box [23].
2. *Evaluate the criteria for good measurement properties using quality criteria*: The measurement properties of each included instrument will be evaluated using the COSMIN recommended criteria for good measurement properties, as outlined in Table 2 [15, 22, 27].

**Table 2:** COSMIN recommended criteria for good measurement properties

Measurement property	Rating	Criteria
Structural validity	+	**Classical Test Theory (CTT)** Confirmatory factor analysis: Comparative Fit Index or Tucker Lewis Index or comparable measure > 0.95 OR root mean square error of approximation < 0.06 OR standardised root mean residuals < 0.08 **Item Response Theory (IRT)/Rasch** No violation of unidimensionality: Comparative Fit Index or Tucker Lewis Index or comparable measure > 0.95 OR root mean square error of approximation < 0.06 OR standardised root mean residuals < 0.08 AND No violation of local independence: residual correlations among the items after controlling for the dominant factor < 0.20 OR Q3’s < 0.37 AND No violation of monotonicity: adequate looking graphs OR item scalability > 0.30 AND Adequate model fit: IRT: *χ*2 > 0.001 Rasch: infit and outfit mean squares ≥ 0.5 and ≤ 1.5 OR Z-standardized values > − 2 and < 2
	?	**CTT**: not all information for ‘+’ reported **IRT/Rasch**: model fit not reported
	−	Criteria for ‘+’ not met
Internal consistency	+	At least low evidence for sufficient structural validity AND Cronbach’s alpha(s) ≥ 0.70 for each unidimensional scale or subscale
	?	Criteria for ‘At least low evidence for sufficient structural validity’ not met
	−	At least low evidence for sufficient structural validity AND Cronbach’s alpha(s) < 0.70 for each unidimensional scale or subscale
Reliability	+	Interclass Correlation Coefficient (ICC) or weighted kappa ≥ 0.70
	?	ICC or weighted kappa not reported
	−	ICC or weighted kappa < 0.70
Measurement error	+	Smallest detectable change (SDC) or limits of agreement (LoA) < minimal important change (MIC)
	?	MIC not defined
	−	SDC or LoA > MIC
Hypotheses testing for construct validity	+	The result is in accordance with the hypothesis
	?	No hypothesis defined (by the review team)
	−	The result is not in accordance with the hypothesis
Cross-cultural validity/measurement invariance	+	No important differences found between group factors (e.g. age and language) in multiple group factor analysis OR no important differential item functioning (DIF) for group factors (McFadden’s *R* < 0.02)
	?	No multiple group factor analysis OR DIF analysis performed
	−	Important differences between group factors OR DIF was found
Criterion validity	+	Correlation with gold standard ≥ 0.70 OR area under the curve (AUC) ≥ 0.70
	?	Not all information for ‘+’ reported
	−	Correlation with gold standard < 0.70 OR AUC < 0.70
Responsiveness	+	The result is in accordance with the hypothesis OR AUC ≥ 0.70
	?	No hypothesis defined (by the review team)
	−	The result is not in accordance with the hypothesis OR AUC < 0.70

The reported findings for each measurement property will be compared with the predetermined criteria described in Table 2, and the adequacy of results of each study will be rated as sufficient (+), intermediate (?) or insufficient (−).

3. *Summarising the evidence*: The results retrieved from 1 and 2 above will be evaluated for consistency across studies. If the results are consistent they will be summarised, compared against the criteria for good measurement properties and deemed as (+) sufficient, (−) insufficient, (±) inconsistent or intermediate (?). As per COSMIN guidance [22], we will explore any inconsistency in results. If an explanation is found, an overall rating of the instrument will be provided for several subgroups. The subgroups of interest include ethnicity, religion, sexual orientation, delivery type, age of participants (e.g. under 18), type of country (developing vs. developed), setting (public hospital, private clinic, midwife led, etc.), multiple vs. singleton pregnancy and nulliparous vs. multiparous women.

### Additional evaluation

As per the stated study objectives, additional evaluations will be made on the following:

1. *Items included within each survey instrument*:

The purpose of categorising items that are included within each instrument is to evaluate what aspects of care are being measured as it has been acknowledged that a limitation of survey instruments, and possibly the reason why there has been a proliferation of such instruments in recent times, is that feedback is provided only on aspects of care that are included specifically [9]. The inclusion of this objective allows for the identification of the most commonly used items within survey instruments that encompass the entire maternity care process and will highlight gaps or inconsistencies across the domains of all instruments. A data extraction form has been developed to standardise the collection of information related to the items included within each survey instrument and to aid analysis (Table 3). All relevant information that is available within the included studies will be extracted as direct quotes to populate the data extraction form

**Table 3:** Data extraction form

General information	Original author
	Title
	Journal
	Year
	Country of origin
	Language and available translations
	Background of the jurisdictions healthcare
	Study design
	Study aim
Instrument details/items included	Outcome measure
	Purpose/use
	Number of domains
	Number of items
	Structure of domains and items
	List of complete bank of items included^a^
	Scale design (type of scale, e.g Likert, no. of points of scale)
	Target population—inclusion and exclusion criteria

^a^Bank of items included within each survey instrument to be recorded within an additional file under the headings antenatal, intranatal, postnatal and misc

### Planned methods of analysis

General information and instrument detail will be summarised in tabular format. The reasons for excluding studies at full text will also be reported in tabular format. The analysis of each retrieved item pool and the use of this in later phases of the project will be published separately.

The methodological quality and results of each instrument will be compared using a structured narrative synthesis. Additionally, an overview of the combined results of measurement properties will be summarised in tabular format as described above under the subheading ‘Summarising the evidence’. This combination of analysis will allow for recommendations to be made on the use, adaptation and development of future survey instruments measuring women’s experiences of their maternity care.

## Discussion

The value of survey instruments that measure women’s experiences of their maternity care has been recognised internationally with many countries employing the use of such instruments to inform policy and practice. In influencing policy and practice, the data arising from these instruments is used to direct limited resources within maternity services. As such, the development of instruments must be methodologically sound and the instrument itself fit for the purpose and context in which it is used to ensure the resulting data is credible. This systematic review will serve as a guide for choosing the most appropriate existing instruments to use or adapt so that they are fit for purpose, in addition to informing the development of new instruments. This review is timely, not only as it fills a gap in the current literature, but also because the use and development of such instruments is increasing internationally.

## Data Availability

Not applicable.

## Abbreviations

AUC: Area under the curve
CINAHL: Cumulative Index of Nursing and Allied Health Literature
COSMIN: COnsensus-based Standards for the selection of health Measurement Instruments
CTT: Classical Test Theory
DIF: Differential item functioning
EMBASE: Excerpta Medica dataBASE
ICC: Interclass correlation coefficient
IRT: Item Response Theory
LoA: Limits of agreement
MEDLINE: MEDLINE is the online counterpart to MEDLARS MEDical Literature Analysis and Retrieval System
MIC: Minimal important change
PRISMA-P: Preferred Reporting Items for Systematic Reviews and Meta-Analyses- Protocols
PRISMA: Preferred Reporting Items for Systematic Reviews and Meta-Analyses
PROM: Patient-reported outcome measure
PROSPERO: International Prospective Register of Systematic Reviews
SDC: Smallest detectable change
